# HIV-1 molecular transmission clusters in nine European countries and Canada: association with demographic and clinical factors

**DOI:** 10.1186/s12916-018-1241-1

**Published:** 2019-01-08

**Authors:** Dimitrios Paraskevis, Apostolos Beloukas, Kostantinos Stasinos, Nikos Pantazis, Carmen de Mendoza, Norbert Bannert, Laurence Meyer, Robert Zangerle, John Gill, Maria Prins, Antonella d’Arminio Montforte, Anne-Marte Bakken Kran, Kholoud Porter, Giota Touloumi, on behalf of the CASCADE collaboration of EuroCoord

**Affiliations:** 1 Department of Hygiene, Epidemiology and Medical Statistics, Medical School, National and Kapodistrian University of Athens, 75 Mikras Asias Street, 115 27 Athens, Greece; 2 Institute of Infection and Global Health, University of Liverpool, Ronald Ross Building, 8 West Derby Street, Liverpool, L69 7BE UK; 3 Department of Biomedical Sciences, School of Health Sciences, University of West Attica, Agiou Spiridonos Str (Campus 1), 12243 Athens, Greece; 4 Department of Internal Medicine, Puerta de Hierro Research Institute and University Hospital, Alle Manuel de Falla, 1, 28222 Madrid, Majadahonda Spain; 5 Robert Koch Institute, Nordufer 20, 13353 Berlin, Germany; 6 Inserm, CESP U1018, Univ Paris-Sud, Department of Epidemiology and Population Health, APHP, Hôpital Bicêtre, 78 Rue du Général Leclerc, 94270 Le Kremlin-Bicêtre, France; 7 Department of Dermatology and Venerology, Innsbruck Medical University, Anichstraße 35, 6020 Innsbruck, Austria; 8 Department of Microbiology, Immunology and Infectious Diseases (MIID), University of Calgary, 269 Heritage Medical Research Building, 24 Ave NW, Calgary, Alberta Canada; 9 Academic Medical Center, University of Amsterdam, Netherlands and Department of Infectious Diseases, Amsterdam Infection and Immunity Institute, Spui 21, 1012 WX Amsterdam, Netherlands; 10 Department of Health Sciences, University of Milan, Via di Rudinì, 8, 20142 Milan, Italy; 11 Department of Microbiology, Oslo University Hospital, OUS HF Rikshospitalet, Postboks 4950 Nydalen, 0424 Oslo, Norway; 12 Institute of Clinical Medicine, University of Oslo, Sognsvannsveien 20, Rikshospitalet, 0372 Oslo, Norway; 13 University College London Institute for Global Health, Institute of Child Health, 3rd floor, 30 Guilford Street, London, WC1N 1EH UK

**Keywords:** Transmission networks, Regional epidemics, Phylogenies, Clusters, HIV epidemic, HIV, Molecular epidemiology

## Abstract

**Background:**

Knowledge of HIV-1 molecular transmission clusters (MTCs) is important, especially in large-scale datasets, for designing prevention programmes and public health intervention strategies. We used a large-scale HIV-1 sequence dataset from nine European HIV cohorts and one Canadian, to identify MTCs and investigate factors associated with the probability of belonging to MTCs.

**Methods:**

To identify MTCs, we applied maximum likelihood inferences on partial *pol* sequences from 8955 HIV-positive individuals linked to demographic and clinical data. MTCs were defined using two different criteria: clusters with bootstrap support >75% (phylogenetic confidence criterion) and clusters consisting of sequences from a specific region at a proportion of >75% (geographic criterion) compared to the total number of sequences within the network. Multivariable logistic regression analysis was used to assess factors associated with MTC clustering.

**Results:**

Although 3700 (41%) sequences belonged to MTCs, proportions differed substantially by country and subtype, ranging from 7% among UK subtype C sequences to 63% among German subtype B sequences. The probability of belonging to an MTC was independently less likely for women than men (OR = 0.66; *P* < 0.001), older individuals (OR = 0.79 per 10-year increase in age; *P* < 0.001) and people of non-white ethnicity (OR = 0.44; *P* < 0.001 and OR = 0.70; *P* = 0.002 for black and ‘other’ versus white, respectively). It was also more likely among men who have sex with men (MSM) than other risk groups (OR = 0.62; *P* < 0.001 and OR = 0.69; *P* = 0.002 for people who inject drugs, and sex between men and women, respectively), subtype B (ORs 0.36–0.70 for A, C, CRF01 and CRF02 versus B; all *P* < 0.05), having a well-estimated date of seroconversion (OR = 1.44; *P* < 0.001), a later calendar year of sampling (ORs 2.01–2.61 for all post-2002 periods versus pre-2002; all *P* < 0.01), and being naïve to antiretroviral therapy at sampling (OR = 1.19; *P* = 0.010).

**Conclusions:**

A high proportion (>40%) of individuals belonged to MTCs. Notably, the HIV epidemic dispersal appears to be driven by subtype B viruses spread within MSM networks. Expansion of regional epidemics seems mainly associated with recent MTCs, rather than the growth of older, established ones. This information is important for designing prevention and public health intervention strategies.

**Electronic supplementary material:**

The online version of this article (10.1186/s12916-018-1241-1) contains supplementary material, which is available to authorized users.

## Background

HIV remains a major global public health issue with an estimated 36.7 million people living with HIV (PLWH) by the end of 2016 [1]. Since the late 1990s, the progressive availability and success of combination antiretroviral therapy has reduced the risk of opportunistic infections and malignancies in PLWH, remarkably decreasing morbidity and mortality [1]. Global efforts to strengthen HIV treatment programmes have not only transformed HIV to a manageable lifelong disease, but also constitute the most effective strategy for preventing onward transmission of infection and, thus, expansion of the epidemic [2, 3]. Nonetheless, the annual number of new HIV infections remains high, with 1.8 million new infections in 2016, and the pace of decline is far too slow to reach global targets [1, 4, 5]. Thus, global HIV prevention and treatment programmes must be guided by information on the sources of new infections and factors driving epidemic maintenance and growth.

The study of the HIV epidemic by molecular phylogenetics has been revolutionised by tools to assess the structure and dispersal of mainly local or regional epidemics [6–8]. When viruses retain a high degree of genetic similarity relative to others, one can assume that their corresponding hosts are related by one or more recent transmission events. HIV-1 is well suited for these analyses because of its high nucleotide substitution rate, which allows the observation of evolutionary changes over a short time period [9, 10]. Clustered sequences can infer putative transmission networks, and phylogenetic cluster analysis, combined with epidemiological and demographic data, can help identify the factors underlying the growth of both regional and global epidemics [11–13]. Therefore, large-scale analyses of HIV-1 phylogenies to extract meaningful epidemiological information for evolutionary relationships and transmission history are feasible [2, 3]. Such studies are important to identify the transmission of drug-resistant variants and to design prevention programmes and public health intervention strategies [2, 3, 13–15].

In this study, we use a large HIV-1 sequence dataset of HIV cohorts from nine European countries and one from Canada to undertake molecular phylogenetic analyses to identify and characterise molecular transmission clusters (MTCs). We also examine the likely impact of clinical and demographic factors on regional phylogenetic clustering.

## Methods

### Patient data

As part of the EuroCoord collaboration [16], HIV-1 sequence data linked to epidemiological and clinical data were available for 9265 of approximately 32,000 individuals enrolled by September 2014 into one of 10 cohorts from France, Germany, Greece, Italy, the Netherlands, Norway, UK, Austria, Spain and Canada. A subset of these data was from individuals with well-estimated HIV seroconversion dates (thereafter termed ‘seroconverters’) from the CASCADE (Concerted Action on SeroConversion to AIDS and Death in Europe) collaboration database.

All patients enrolled into the study gave their written informed consent.

### HIV-1 sequences dataset

A pooled initial dataset of 18,655 HIV-1 sequences were available, including protease and partial reverse transcriptase (RT) sequences, alone or combined, and some integrase sequences. These were merged into a dataset of 8955 partial *pol* sequences (i.e., protease and partial RT). Duplicates were excluded using the online tool ElimDupes [17], resulting in one sequence per individual. All study sequences were generated as part of routine clinical resistance testing at the participating sites using standard (Sanger) sequencing procedures.

### HIV-1 subtyping and reference datasets

Subtyping was performed using the online automated subtyping tools COMET (COntext-based Modeling for Expeditious Typing) [18] and REGAv.2.0 [19]. Un-subtyped and undetermined sequences were phylogenetically subtyped as previously described [20].

MTCs were identified using a large sample of subtype-specific reference sequences from the Los Alamos HIV-1 sequence database [21] in separate subtype-specific alignments as explained below. Analyses were conducted only for the most prevalent subtypes, i.e. A–D, F and G, and the circulating recombinant forms (CRF) CRF01_AE and CRF02_AG; other subtypes with low proportions in the study dataset (< 0.6%) were not analysed further. Reference datasets for all non-B subtypes, CRF01_AE and CRF02_AG included all *pol* sequences (protease and partial RT) that were publicly available at the time of analysis. The number of reference sequences used per subtype was A, 3782; C, 6581; D, 1216; F, 837; G, 1026; CRF01_AE, 2696; and CRF02_AG, 2622. Given the large numbers of subtype B in the HIV Los Alamos database, a final reference dataset of 14,946 out of 42,470 (34.1%) available sequences randomly resampled from different geographic areas and sampling dates was used. All duplicate sequences were excluded prior to analysis.

Study sequences and subtype-specific reference sequences for each subtype and CRF were aligned separately using the MUSCLE programme in subtype-specific alignments [22]. Alignments were manually trimmed using MEGA 6.0 [23] and mutation sites described in the International Antiviral Society of the USA’s (IAS-USA) 2017 published list of Drug Resistance Mutations in HIV-1 [24] were excluded from all datasets prior to any analyses.

### Identification of molecular transmission clusters

A two-step analysis approach was followed. Initially, maximum likelihood (ML) phylogenetic inference and bootstrap analysis, as implemented in the RAxML-HCP2 tool, was performed [25]. ML phylogenies were estimated using the general time-reversible substitution model with gamma rate heterogeneity among sites. MTCs were defined as those clusters with ≥ 2 sequences from the same country having bootstrap support greater than 75% (phylogenetic confidence criterion) and those consisting of sequences from a specific area at a proportion greater than 75% (geographic criterion) compared to the total number of sequences within the cluster. Subsequently, an additional confirmatory analysis was performed for the clusters that initially received lower bootstrap support values, namely those between 50% and 75%. Briefly, the consensus sequence for each cluster was estimated, then, using BLAST [26], the 100 most relevant sequences to the consensus were downloaded and used for the confirmatory analysis. Phylogenetic analysis was performed using the Bayesian method with the general time-reversible substitution model with Γ-distributed rate, as implemented in MrBayes 3.2.2 [27]. The confirmatory analysis was performed on a subset of clusters, namely those comprising ≥ 5 sequences fulfilling the geographic criterion, receiving support between 50% and 75%. The Markov chain Monte Carlo method was run for 2.2x10^6^ generations (burnin was set to 2x10^5^ generations; 10%), with four chains per run. This was sampled every 1000 steps and was checked for convergence, as previously described [28].

### Statistical analysis

Demographic and clinical data are summarised using median and interquartile ranges (for continuous variables), or absolute and relative frequencies (for categorical variables). Simple comparisons of the relevant distributions across different levels of other categorical variables are based on chi-square tests for categorical variables, or non-parametric (Mann–Whitney, Kruskal–Wallis) tests. Associations of the probability of belonging to an MTC with various demographic and clinical characteristics (sex, age, mode of transmission, sampling date, subtype, ethnic group, antiretroviral therapy (ART) experience, country, known seroconversion) were investigated using logistic regression models. All variables were used as categorical variable, except for age, which was used as a continuous variable because its effects did not deviate significantly from linearity. As a sensitivity analysis, the final multivariable logistic regression model was also fitted to subsets of the full dataset, excluding data from each of the three smallest cohorts (the Netherlands, Greece, and France), or all of them simultaneously.

## Results

### Study population

Overall, 8955 of 9265 (96.7%) individuals with HIV-1 protease/partial RT sequences and matched demographic and clinical data were enrolled in the study. Included individuals were predominantly male (6959/8959; 77.7%) and from the ‘men who have sex with men’ (MSM) risk group (4980/8955; 55.6%). The majority of included sequences originated from Spain (n = 1978), followed by the UK (n = 1559) and Germany (n = 1542); more than 50% of the data in the study dataset came from these three countries (see Additional file 1: Table S1). Almost one-third (n = 3050; 34.1%) of the study population had well-estimated seroconversion dates. Demographic and clinical characteristics of the corresponding individuals are presented in Table 1.

**Table 1:** Demographic and clinical characteristics of the study population according to whether or not they belong to a molecular transmission cluster

	Non-clustered n=5,255	Clustered n=3,700	Overall N=8,955	
	N (%)	N (%)	N (%)	*P*-value
Sex				< 0.001
Male	3805 (72)	3154 (85)	6959 (78)	
Female	1120 (21)	331 (9)	1451 (16)	
Unknown	330 (6)	215 (6)	545 (6)	
Risk group				< 0.001
MSM	2423 (46)	2557 (69)	4980 (56)	
PWID	692 (13)	246 (7)	938 (11)	
MSW	1510 (29)	577 (16)	2087 (23)	
Haemophiliacs	10 (<1)	2 (<1)	12 (<1)	
Other – unknown	620 (12)	318 (9)	938 (11)	
Ethnicity				< 0.001
White	2434 (46)	1419 (38)	3853 (43)	
Black	377 (7)	72 (2)	449 (5)	
Other	377 (7)	160 (4)	537 (6)	
Unknown	2067 (39)	2049 (55)	4116 (46)	
Country				< 0.001
Canada	558 (11)	383 (10)	941 (11)	
France	18 (<1)	5 (<1)	23 (<1)	
Germany	641 (12)	901 (24)	1542 (17)	
Greece	28 (<1)	7 (<1)	35 (<1)	
Italy	909 (17)	188 (5)	1097 (12)	
Netherlands	51 (1)	7 (<1)	58 (<1)	
Norway	399 (8)	226 (6)	625 (7)	
UK	975 (19)	584 (16)	1559 (17)	
Austria	687 (13)	410 (11)	1097 (12)	
Spain	989 (19)	989 (27)	1978 (22)	
Subtype				< 0.001
B	4195 (80)	3350 (91)	7545 (84)	
C	352 (7)	81 (2)	433 (5)	
A	192 (4)	68 (2)	260 (3)	
CRF01_AE	156 (3)	36 (1)	192 (2)	
CRF02_AG	224 (4)	89 (2)	313 (4)	
D	42 (<1)	11 (0)	53 (<1)	
G	45 (<1)	24 (0)	69 (<1)	
F	49 (<1)	41 (1)	90 (1)	
Sampling date				< 0.001
1987–2002	1484 (28)	451 (12)	1935 (22)	
2003–2006	1254 (24)	984 (27)	2238 (25)	
2007–2008	1135 (22)	1062 (29)	2197 (25)	
2009–2011	1004 (19)	956 (26)	1960 (22)	
Not available	378 (7)	247 (7)	625 (7)	
Seroconversion date				< 0.001
1981–1996	607 (12)	160 (4)	767 (9)	
1997–2003	497 (10)	373 (10)	870 (10)	
2004–2006	261 (5)	410 (11)	671 (8)	
2007–2011	299 (6)	443 (12)	742 (8)	
Not known	3591 (68)	2314 (63)	5905 (66)	

Abbreviations: *MSM* men who have sex with men, *PWID* people who inject drugs, *MSW* men who have sex with men and women

**Table 2:** Proportion of sequences belonging to a molecular transmission cluster (MTC) by cohort country and HIV-1 subtype

Country	A		B		C		CRF01_AE		CRF02_AG		Other (D, F, G)		Overall		
	MTC N (%)	Not MTC N (%)	MTC N (%)	Not MTC N (%)	MTC N (%)	Not MTC N (%)	MTC N (%)	Not MTC N (%)	MTC N (%)	Not MTC N (%)	MTC N (%)	Not MTC N (%)	MTC N (%)	Not MTC N (%)	Total N (%)
Canada	7 (30.4)	16 (69.6)	332 (47.4)	368 (52.6)	29 (18.2)	130 (81.8)	4 (36.4)	7 (63.6)	6 (24.0)	19 (76.0)	5 (21.7)	18 (78.3)	383 (40.7)	558 (59.3)	941 (100.0)
France			5 (29.4)	12 (70.6)						6 (100.0)			5 (21.7)	18 (78.3)	23 (100.0)
Germany	4 (12.9)	27 (87.1)	869 (62.8)	514 (37.2)	5 (20.0)	20 (80.0)	7 (17.5)	33 (82.5)	14 (29.8)	33 (70.2)	2 (12.5)	14 (87.5)	901 (58.4)	641 (41.6)	1542 (100.0)
Greece	2 (16.7)	10 (83.3)	5 (23.8)	16 (76.2)						1 (100.0)		1 (100.0)	7 (20.0)	28 (80.0)	35 (100.0)
Italy	4 (57.1)	3 (42.9)	164 (16.1)	854 (83.9)		10 (100.0)		3 (100.0)	4 (18.2)	18 (81.8)	16 (43.2)	21 (56.8)	188 (17.1)	909 (82.9)	1097 (100.0)
Netherlands			7 (12.1)	51 (87.9)									7 (12.1)	51 (87.9)	58 (100.0)
Norway	6 (14.3)	36 (85.7)	165 (43.5)	214 (56.5)	25 (23.6)	81 (76.4)	13 (28.9)	32 (71.1)	9 (27.3)	24 (72.7)	8 (40.0)	12 (60.0)	226 (36.2)	399 (63.8)	625 (100.0)
UK	4 (13.3)	26 (86.7)	574 (40.1)	856 (59.9)	4 (6.9)	54 (93.1)		14 (100.0)		19 (100.0)	2 (25.0)	6 (75.0)	584 (37.5)	975 (62.5)	1559 (100.0)
Austria	23 (28.1)	59 (72.0)	301 (42.0)	429 (58.0)	21 (18.6)	48 (81.4)	12 (16.2)	62 (83.8)	18 (25.4)	53 (74.7)	35 (49.3)	36 (50.7)	410 (37.4)	687 (62.6)	1097 (100.0)
Spain	18 (54.6)	15 (45.5)	918 (51.0)	881 (49.0)	7 (43.8)	9 (56.3)		5 (100.0)	38 (42.7)	51 (57.3)	8 (22.2)	28 (77.8)	989 (50.0)	989 (50.0)	1978 (100.0)
Total	68 (26.2)	192 (73.9)	3350 (44.4)	4195 (55.6)	81 (18.7)	352 (81.3)	36 (18.8)	156 (81.3)	89 (28.4)	224 (71.6)	76 (35.9)	136 (64.2)	3700 (41.3)	5255 (58.7)	8955 (100.0)

Abbreviation: *MTC* Molecular transmission clusters

### Subtype analysis

Almost 85% of sequences were of the B subtype (7545; 84.3%), followed by subtypes C (433; 4.8%) and A (260; 2.9%). Among the recombinants, the most frequent were CRF02_AG (313; 3.5%) and CRF01_AE (192; 2.1%) (see Additional file 1: Table S1). All other subtypes (F, D and G) and other CRFs were much less common at 1% or below (data not shown). Notably, the distribution of subtypes differed significantly by country. In the study dataset, the proportion of subtype B sequences ranged from 60% in Greece to 100% in the Netherlands. Greek sequences in the study dataset had the highest proportion (34.3%; 12/35) of subtype A sequences. High proportions of subtype C were found in the sequences from Canada (16.9%; 159/941) and Norway (17.0%; 106/625), while the highest proportion of CRF02_AG (27.3%; 6/23) was in the French data. The distribution of subtypes, according to cohort country and risk group, are shown in Additional file 1: Table S1.

### Identification of MTCs

**Fig. 1:**
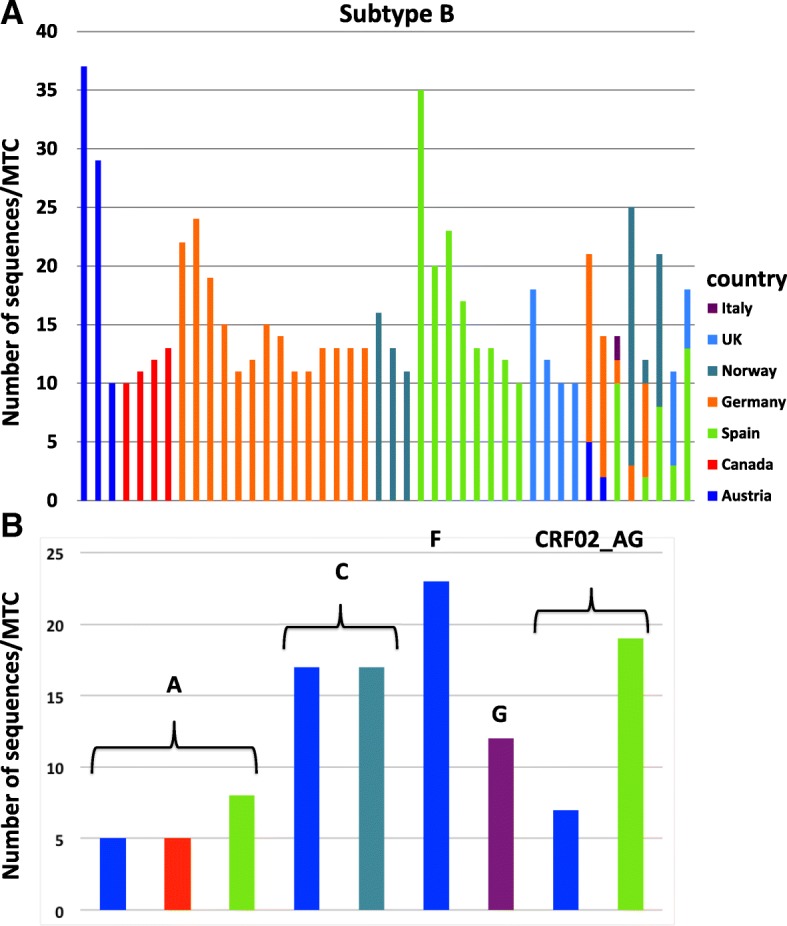
Number of sequences and cohort country for the largest molecular transmission clusters (MTCs) consisting of ≥ 10 sequences for subtype B (**a**) and of ≥ 5 non-B and CRF_02_AG sequences (**b**)

After the first analysis step (ML phylogenetic inference), we identified 1125 putative MTCs comprising sequences from the same country. Of these, 156 (13.9%), 93 (8.3%) and 876 (77.9%) had bootstrap support of 50–65%, 66–75% and >75%, respectively. Therefore, 77.9% of all clusters fulfilled both criteria for MTCs in the first step (see Additional file 2: Table S2). Each of the 1125 MTCs consisted of 2–37 sequences from unique individuals, although most (58%; n = 653) were small networks of two individuals each. The largest MTC was for subtype B and included 37 sequences from Austria. Large MTCs consisting of ≥ 12 sequences were also identified for subtypes C, G, F and CRF02. Finally, the biggest nationally mixed MTC included 25 subtype B sequences from Norway (n = 22) and Germany (n = 3) (Fig. 1).

Many subtype B clusters (n = 230) fulfilled the geographic criterion for MTCs, but had bootstrap support below the threshold of 75% (see Additional file 2: Table S2). Fifty-eight of those with ≥ 5 sequences underwent the confirmatory analysis. This showed that initial clustering was robust in all 58 subtype B MTCs; 40/148 (27.0%) with bootstrap support of 50–65% and 18/82 (22.0%) with bootstrap support of 66–75% always receiving a posterior probability support greater than 0.95.

**Fig. 2:**
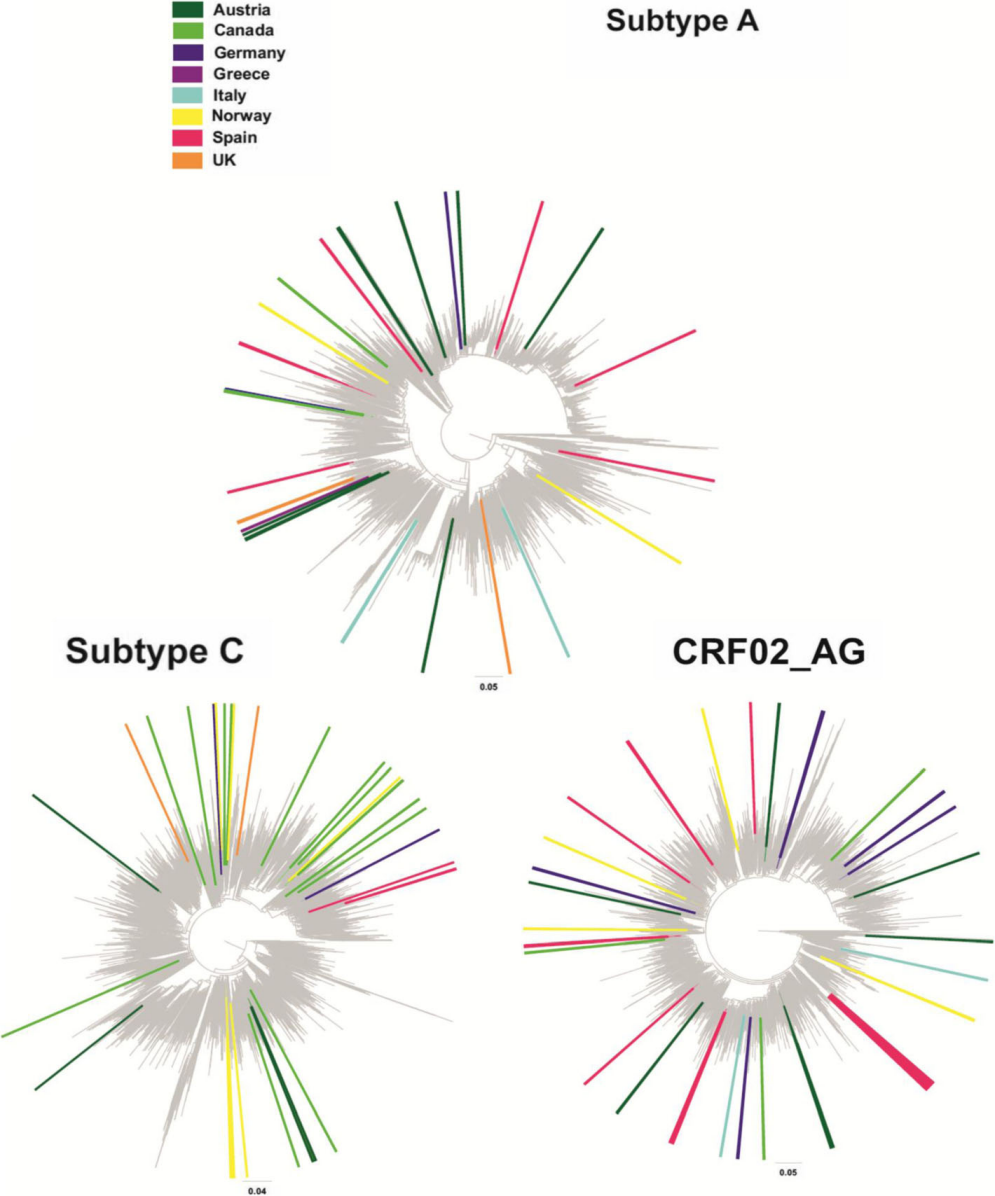
Clustering of HIV-1 sequences within the biggest molecular transmission clusters (MTCs) for subtypes A and G and CRF02_AG

After initial and confirmatory analyses, we identified that 3700/8955 (41.3%) sequences belonged to MTCs. Specifically, for subtype B, the sequences clustered in MTCs ranged from 12% in the Netherlands to 63% in Germany, while for subtype C, the proportion included in MTCs ranged between 7% for the UK and 44% for Spain (Table 2). In Spain, we identified that the highest proportion of clustered sequences belonged to CRF02_AG (38/89, 42.7%) and A (18/33, 54.6%) (Fig. 2). Canadian sequences, with respect to their low numbers, represented the highest percentage of clustered sequences for CRF01_AE (4/11, 36.4%) and subtype D (5/12, 41.7%) (Table 2). Finally, 29/41 (70.7 %) of subtype F sequences from Austria clustered together, including one MTC of 23 sequences and three small clusters of two sequences each, and 12/17 (70.6%) of subtype G sequences from Italy clustered together (Fig. 1b).

More specifically, for subtype B MTCs, 25/833 (3.0%) were nationally mixed MTCs, comprising 231 from 3350 (6.9%) subtype B sequences clustered to MTCs originating from two or three of the following countries: Austria, Germany, Italy, Norway, Spain and UK. Ten out of 25 (40.0%) of these were identified from the initial ML phylogenies, while another 15 (60.0%) were identified after the confirmatory analysis.

### Association of clustering with demographic and clinical factors

Table 3 presents the results from multivariable logistic regression models for the association between the probabilities of belonging to an MTC with other demographic or clinical factors. Women were less likely to belong to an MTC than men (OR = 0.66; 95% CI, 0.56–0.78; *P* < 0.001), as were those of black or other ethnicity than white (black versus white: OR = 0.44, 95% CI, 0.32–0.62, *P* < 0.001; other ethnicity versus white: OR = 0.70, 95% CI, 0.55–0.88; *P* = 0.002). Sequences of subtypes A and C and CRFs CRF01_AE or CRF02_AG were significantly less likely to cluster than subtype B. MSM were more likely to cluster than all other risk groups. Younger age and being ART-naïve at sampling were also associated with increased probabilities of belonging to an MTC.

**Table 3:** Factors associated with the probability of belonging to a molecular transmission cluster: results from a multivariable logistic regression model

Covariate	Odds ratio	95% CI	*P*-value
Sex			
Male ^a^	1		
Female	0.66	(0.56–0.78)	< 0.001
Unknown	0.44	(0.04–5.10)	0.514
Subtype			
B ^a^	1		
CRF02_AG	0.70	(0.53–0.94)	0.016
C	0.51	(0.38–0.69)	< 0.001
A	0.65	(0.48–0.89)	0.007
CRF01_AE	0.36	(0.24–0.54)	< 0.001
Other (D,F,G)	1.04	(0.76–1.42)	0.814
Country			
Germany ^a^	1		
Canada	0.91	(0.66–1.26)	0.584
Spain	0.7	(0.59–0.83)	< 0.001
Norway	0.68	(0.49–0.94)	0.021
Austria	0.68	(0.50–0.92)	0.014
UK	0.5	(0.36–0.69)	< 0.001
Italy	0.39	(0.29–0.53)	< 0.001
France	0.23	(0.06–0.86)	0.028
Netherlands	0.2	(0.09–0.45)	< 0.001
Greece	0.17	(0.07–0.41)	< 0.001
Age at sample date			
Per 10-year increase	0.79	(0.75–0.84)	< 0.001
Sampling date			
1987–2002 ^a^	1		
2003–2006	2.01	(1.67–2.43)	< 0.001
2007–2008	2.38	(1.95–2.91)	< 0.001
2009–2011	2.61	(2.12–3.19)	< 0.001
Seroconverter			
No ^a^	1		
Yes	1.44	(1.23–1.69)	< 0.001
Risk group			
MSM ^a^	1		
PWID	0.62	(0.52–0.74)	< 0.001
MSW	0.69	(0.59–0.80)	< 0.001
Haemophiliacs	0.27	(0.06–1.27)	0.097
Other – unknown	0.55	(0.42–0.72)	< 0.001
Ethnicity			
White ^a^	1		
Black	0.44	(0.32–0.62)	< 0.001
Other	0.70	(0.55–0.88)	0.002
Unknown	0.91	(0.72–1.17)	0.467
ART-naïve at sampling			
No ^a^	1		
Yes	1.19	(1.04–1.35)	0.010

Abbreviations: *MSM* men who have sex with men, *PWID* people who inject drugs, *MSW* men who have sex with men and women

^a^ Reference category

A trend was observed for an increased probability of clustering in individuals who contributed samples in more recent calendar periods and in PLWH with well-estimated seroconversion dates. Finally, clustering probabilities differed by cohort country, with higher probabilities observed in Germany and Canada followed by Spain. Individuals followed up in Greece, the Netherlands and France had the lowest probabilities of belonging to an MTC. Repeating the analysis after exclusion of participants belonging to one or all of these small cohorts yielded estimates with negligible differences compared with those of the main analysis.

## Discussion

Phylogenetic analyses of ~9000 HIV-1 sequences revealed that >40% of them belonged in MTCs. While this observation is consistent with other reports of HIV-1 epidemic dispersal in these countries [29–34], our study is among the first to investigate the structure of these regional HIV-1 phylogenies in greater detail, using a large-scale sequence dataset, dense reference sequence sampling and associating multiple clinical and demographic factors with the dispersal of MTCs.

An additional strength of this study is that all the available sequences of non-B and CRF subtypes deposited in the HIV Los Alamos database were used as reference sequences for phylogenetic analysis. For subtype B, we used more than one-third of the publicly available references sequences (14,946 of 42,470; 34.1%) after random selection representative of the global subtype B epidemic. Finally, MTCs were identified as those clustered sequences fulfilling both phylogenetic (bootstrap value > 75% or posterior probability support > 0.95) and geographic criteria (75% of clustered sequences from the same region). To date, there is no consensus on the methodology used to infer HIV-1 transmission clusters [35]. In our study we used both geographic and phylogenetic criteria and a large number of globally sampled reference sequences to identify MTCs.

Not surprisingly for these 10 countries, subtype B was the most prevalent subtype in this dataset (84.3%), followed by subtypes C (4.8%), CRF02_AG (3.5%), A (2.9%) and CRF01_AE (2.1%), which is consistent with previously reported data [29, 36, 37]. Notably, the probability of clustering in an MTC was significantly higher among subtype B than non-B sequences (ORs, CRF02_AG = 0.70, A = 0.65, C = 0.51 and CRF01_AE = 0.36; range of *P*-values 0.001–0.016) (Table 3). Some studies have noted differences in the biological properties of HIV-1 subtypes [38, 39], but there is no conclusive evidence that certain subtypes are more infectious or have higher transmissibility than others. This is most likely because of the high prevalence of subtype B infections in individuals enrolled in the study cohorts versus non-B subtypes and recombinants, rather than differences in the transmissibility and infectivity of subtype B viruses. It was the subtype B form of HIV-1 that was introduced to Western Europe and this remains the most prevalent subtype across Europe [29, 36]. However, infections with non-B subtypes are more common among individuals from highly endemic areas, with sex between men and women being the predominant HIV risk factor. The only exceptions in Western Europe are Greece and Portugal, where subtypes G and A have spread successfully among the local populations [29, 40]. Given the characteristics of the spread of these HIV-1 subtypes throughout Western Europe, the finding that subtype B infections have a higher probability of belonging to an MTC reflects that local populations are more likely to be infected within their country (e.g., through regional networks). This hypothesis is further supported by the differences across ethnic groups. In all comparisons, samples from people of white ethnicity were much more likely to contain sequences belonging to MTCs than others (P < 0.001 in all cases). These findings suggest that differences in the probability of belonging to an MTC are likely to be associated with the fact that residents of each country are more closely linked with each, rather than the fact that they are infected with subtype B per se. In other words, if another subtype, such as C, was dominant in Europe, we would probably observe a similar pattern, but with subtype C rather than B. To date, non-B infections in Western Europe (except for Greece and Portugal) are detected either as single lineages – not grouped with others from the same area, or forming small clusters of few sequences [29, 41]. Our study highlights that non-B subtypes have not been associated with widespread epidemics in Europe, but in some countries there is some evidence for regional expansion [20, 41, 42].

The subtype B epidemic was first described in the MSM population, but was spread among PWID soon afterwards [43]. We also found that the MSM population was more likely to belong to MTCs than heterosexuals, PWID and haemophiliacs, suggesting that the MSM population has a greater chance of transmitting HIV between their members (Table 3). Others have also confirmed this trend [13, 44]. With respect to our findings, there may be a higher prevalence of HIV in this group, a higher probability of HIV transmission through MSM practices or more risky behaviour [13, 44]. The probability of clustering was also higher among younger and ART-naïve individuals, reflecting that the younger age group may engage in more risky behaviour and has higher HIV-RNA levels [11].

Finally, the probability of belonging to an MTC differed by cohort country, with higher probabilities observed in Germany and Canada, followed by Spain (Table 3). Since nearly 50% of study sequences were from the three countries with the highest probabilities (namely Spain, UK and Germany), these observed higher probabilities might be explained by the regional expansion of local epidemics [20, 30, 34].

There are several limitations to this study, as in all molecular epidemiological studies. Firstly, the findings may be distorted by the sampling method used. For instance, in all cohorts, there were more sequences available with more recent sampling dates. Significantly reduced sampling from Greece, France and the Netherlands may have biased our results. To minimise the effect of bias, we used a) highly homogenous inclusion criteria; b) a large-scale sequence cohort dataset and c) a large number of reference sequences (>34% of all available for subtype B and 100% for all other subtypes and CRFs analysed) to infer the fine structure of regional epidemics and dispersal networks. Furthermore, clustering definition of sequences uses both phylogenetic and geographic criteria, enabling higher sensitivity for the identification of MTCs. Although we used stricter definitions for networks, the current definition remains credible because it has been confirmed by Bayesian analysis [28, 45, 46]. Finally, to avoid sampling bias – especially given the lower numbers of sequences from the Greek, French and Dutch cohorts – we repeated the multivariable analysis after excluding participants belonging to one of these three small cohorts. Results of this repeated analysis yielded estimates with negligible differences compared with the main analysis.

We found that sequences from samples from individuals with well-estimated seroconversion dates and more recent sampling dates had a higher probability of belonging to MTCs in the specific regional cohorts. Given improvements in the depth of sampling and efficiency of sequencing, larger and more complete HIV-1 sequence datasets are now available. This suggests that some of the increase in the regional MTCs might, at least in part, be attributed to better capturing of recent transmission events. This is in line with previous findings, in which recently infected patients were found to be crucial in the spread of the HIV epidemic [8, 11]. Thus, prevention measures should specifically target these newer MTCs of specific risk groups. The public health implications of such findings, including treatment strategies, are of special interest.

## Conclusion

Using a large-scale dataset comprising protease and partial RT sequences from unique patients from nine European countries and Canada, which were linked to demographic and clinical data, we identified that a high proportion (>40%) of PLHIV belong to an MTC. The epidemic appears to be driven by subtype B viruses spreading among young people in the MSM population. We also found that the recent increase in regional epidemics might, at least in part, be attributed to recent transmission clusters and not the growth of older, established clusters. This finding is in line with recent observations that recently infected patients are crucial in spreading the HIV-1 epidemic and is of significant importance for designing prevention public health intervention strategies.

## Additional files

Additional file 1: **Table S1.** HIV-1 subtype distribution by cohort country and risk group. (DOCX 25 kb)

Additional file 2: **Table S2.** Bootstrap support values of MTCs identified with ML phylogenies. (DOCX 22 kb)

Additional file 3: CASCADE and EuroCoord acknowledgements, and list of ethics committees. (DOCX 20 kb)

Additional file 4: Study sequences. (XLS 7720 kb)

## Abbreviations

ART: antiretroviral therapy
CRF: circulating recombinant form
MTC: molecular transmission cluster
ML: maximum likelihood
MSM: men who have sex with men
PLWH: people living with HIV
RT: reverse transcriptase
