# Supplementary material for: HIV-1 molecular transmission clusters in nine European countries and Canada: association with demographic and clinical factors
Source: BMC Med. 2019 Jan 8;17:4. doi: 10.1186/s12916-018-1241-1 (PMC6323837; doi:10.1186/s12916-018-1241-1)
Supplement: Supplementary file 1 — Table S1. HIV-1 subtype distribution by cohort country and risk group. (DOCX 25 kb) [file 12916_2018_1241_MOESM1_ESM.docx]

**Table S1.** HIV-1 subtype distribution by cohort country and risk group.

| Subtype | | | | | | | | | | |
| --- | --- | --- | --- | --- | --- | --- | --- | --- | --- | --- |
|  | A | B | C | CRF01_AE | CRF02_AG | D | G | F | Overall |  |
|  | N (%) | N (%) | N (%) | N (%) | N (%) | N (%) | N (%) | N (%) | N (%) | *P*-value |
| Country |  |  |  |  |  |  |  |  |  | < 0.001 |
| Canada | 23 (2) | 700 (74) | 159 (17) | 11 (1) | 25 (3) | 12 (1) | 9 (1) | 2 (< 1) | 941 (11) |  |
| France | 0 (0) | 17 (74) | 0 (0) | 0 (0) | 6 (26) | 0 (0) | 0 (0) | 0 (0) | 23 (< 1) |  |
| Germany | 31 (2) | 1383 (90) | 25 (2) | 40 (3) | 47 (3) | 4 (< 1) | 10 (< 1) | 2 (< 1) | 1542 (17) |  |
| Greece | 12 (34) | 21 (60) | 0 (0) | 0 (0) | 1 (3) | 1 (3) | 0 (0) | 0 (0) | 35 (< 1) |  |
| Italy | 7 (< 1) | 1018 (93) | 10 (< 1) | 3 (< 1) | 22 (2) | 1 (< 1) | 17 (2) | 19 (2) | 1097 (12) |  |
| Netherlands | 0 (0 ) | 58 (100) | 0 (0) | 0 (0) | 0 (0) | 0 (0) | 0 (0) | 0 (0) | 58 (< 1) |  |
| Norway | 42 (7) | 379 (61) | 106 (17) | 45 (7) | 33 (53) | 12 (2) | 7 (1) | 1 (< 1) | 625 (7) |  |
| United Kingdom | 30 (2) | 1430 (92) | 58 (4) | 14 (< 1) | 19 (1) | 4 (< 1) | 1 (< 1) | 3 (< 1) | 1559 (17) |  |
| Austria | 82 (8) | 740 (68) | 59 (5) | 74 (7) | 71 (7) | 14 (1) | 16 (2) | 41 (4) | 1097 (12) |  |
| Spain | 33 (2) | 1799 (91) | 16 (< 1) | 5 (< 1) | 89 (5) | 5 (< 1) | 9 (< 1) | 22 (1) | 1978 (22) |  |
| Risk group |  |  |  |  |  |  |  |  |  | < 0.001 |
| MSM | 38 (< 1) | 4784 (96) | 44 (< 1) | 36 (< 1) | 49 (1) | 4 (< 1) | 5 (< 1) | 20 (< 4) | 4980 (59) |  |
| PWID | 45 (5) | 837 (89) | 17 (2) | 1 (< 1) | 9 (1) | 0 (0) | 13 (1) | 16 (2) | 938 (11) |  |
| MSW | 154 (7) | 1184 (57) | 259 (12) | 145 (7) | 214 (10) | 40 (2) | 48 (2) | 43 (2) | 2087 (25) |  |
| Haemophiliacs | 0 (0) | 11 (92) | 1 (8) | 0 (0) | 0 (0) | 0 (0) | 0 (0) | 0 (0) | 12 (< 1) |  |
| Other – unknown | 15 (4) | 235 (59) | 97 (24) | 7 (2) | 28 (7) | 6 (2) | 2 (< 1) | 11 (2.7) | 401 (5) |  |
| Total | 260 (3) | 7545 (84) | 433 (5) | 192 (2) | 313 (4) | 53 (< 1) | 69 (< 1) | 90 (1) | 8955 (100) |  |
