# Supplementary material for: HIV-1 molecular transmission clusters in nine European countries and Canada: association with demographic and clinical factors
Source: BMC Med. 2019 Jan 8;17:4. doi: 10.1186/s12916-018-1241-1 (PMC6323837; doi:10.1186/s12916-018-1241-1)
Supplement: Supplementary file 2 — Table S2. Bootstrap support values of MTCs identified with ML phylogenies. (DOCX 22 kb) [file 12916_2018_1241_MOESM2_ESM.docx]

**Table S2**. Bootstrap support values of molecular transmission clusters identified with maximum likelihood phylogenies.

| Subtypes | Bootstrap | Clusters N (%) | Total Clusters N (%) |
| --- | --- | --- | --- |
| Subtype A | 50–65% | 1 (4.0) | 25 (2.2) |
|  | 66–75% | 5 (20.0) |  |
|  | > 75% | 19 (76.0) |  |
| Subtype B | 50–65% | 148 (14.7) | 1005 (89.3) |
|  | 66–75% | 82 (8.2) |  |
|  | > 75% | 775 (77.1) |  |
| Subtype C | 50–65% | 1 (3.6) | 28 (2.5) |
|  | 66–75% | 3 (10.7) |  |
|  | > 75% | 24 (85.7) |  |
| Subtype D | 50–65% | 1 (16.7) | 6 (0.5) |
|  | 66–75% | 0 (0) |  |
|  | > 75% | 5 (83.3) |  |
| Subtype F | 50–65% | 1 (11.1) | 9 (0.8) |
|  | 66–75% | 1 (11.1) |  |
|  | > 75% | 7 (77.8) |  |
| Subtype G | 50–65% | 0 (0) | 7 (0.6) |
|  | 66–75% | 1 (13.3) |  |
|  | > 75% | 6 (85.7) |  |
| CRF01_AE | 50–65% | 1 (5.6) | 18 (1.6) |
|  | 66–75% | 0 (0) |  |
|  | > 75% | 17 (94.4) |  |
| CRF02_AG | 50–65% | 3 (11.1) | 27 (2.4) |
|  | 66–75% | 1 (3.7) |  |
|  | > 75% | 23 (85.2) |  |
| Overall | 50–65% | 156 (13.9) | 1125 (100) |
|  | 66–75% | 93 (8.3) |  |
|  | > 75% | 876 (77.9) |  |
