# Supplementary material for: HIV-1 molecular transmission clusters in nine European countries and Canada: association with demographic and clinical factors
Source: BMC Med. 2019 Jan 8;17:4. doi: 10.1186/s12916-018-1241-1 (PMC6323837; doi:10.1186/s12916-018-1241-1)
Supplement: Supplementary file 3 — CASCADE and EuroCoord acknowledgements, and list of ethics committees. (DOCX 20 kb) [file 12916_2018_1241_MOESM3_ESM.docx]

**Additional file 3**

The authors would like to acknowledge the following people involved in the CASCADE collaboration and EuroCoord:

**CASCADE Steering Committee:** Julia Del Amo (Chair), Laurence Meyer (Vice Chair), Heiner C. Bucher, Geneviève Chêne, Osamah Hamouda, Deenan Pillay, Maria Prins, Magda Rosinska, Caroline Sabin, Giota Touloumi.

**CASCADE Co-ordinating Centre:** Kholoud Porter (Project Leader), Ashley Olson, Andrea Cartier, Lorraine Fradette, Sarah Walker, Abdel Babiker.

**CASCADE Clinical Advisory Board:** Heiner C. Bucher, Andrea De Luca, Martin Fisher, Roberto Muga

**CASCADE Collaborators:** Tony Kelleher, David Cooper, Pat Grey, Robert Finlayson, Mark Bloch (PHAEDRA cohort, Australia); Tony Kelleher, Tim Ramacciotti, Linda Gelgor, David Cooper, Don Smith (Sydney AIDS Prospective Study and Sydney Primary HIV Infection cohort); Robert Zangerle (Austrian HIV Cohort Study); John Gill (Canada, South Alberta clinic); Irja Lutsar (Estoniam Tartu Ülikool); Geneviève Chêne, Francois Dabis, Rodolphe Thiebaut (Francem ANRS CO3 Aquitaine cohort); Dominique Costagliola, Marguerite Guiguet (ANRS CO4 French Hospital Database); Philippe Vanhems (Lyon Primary Infection cohort, France); Marie-Laure Chaix, Jade Ghosn (French ANRS CO6 PRIMO cohort); Laurence Meyer, Faroudy Boufassa (ANRS CO2 SEROCO cohort, France); Osamah Hamouda, Karolin Meixenberger, Norbert Bannert, Barbara Bartmeyer (German HIV-1 seroconverter cohort); Anastasia Antoniadou, Georgios Chrysos, Georgios L. Daikos (Greece, AMACS); Giota Touloumi, Nikos Pantazis, Olga Katsarou (Greek haemophilia cohort); Giovanni Rezza, Maria Dorrucci (Italian Seroconversion Study); Antonella d’Arminio Monforte, Andrea De Luca (Italian Cohort Naïve Antiretrovirals); Maria Prins, Ronald Geskus, Jannie van der Helm, Hanneke Schuitemaker (Netherlands, Amsterdam Cohort Studies among homosexual men and drug users); Mette Sannes, Anne Ma Dyrhol-Riise, Anne-Marte Bakken Kran (Norway, Oslo University Hospital cohorts); Magdalena Rosinska (Poland, National Institute of Hygiene); Roberto Muga, Jordí Tor (Spain, Badalona Intravenous Drug User hospital cohort), Patricia Garcia de Olalla, Joan Cayla (Spain, Barcelona Intravenous Drug User Cohort), Julia del Amo, Santiago Moreno, Susana Monge (CoRIS-scv, Spain); Julia Del Amo, Jorge del Romero (Madrid cohort, Spain), Santiago Pérez-Hoyos (Valencia Intravenous Drug User cohort, Spain); Anders Sönnerborg (Swedish InfCare HIV Cohort); Heiner C. Bucher, Huldrych Günthard, Alexandra Scherrer (Swiss HIV Cohort Study); Ruslan Malyuta (Ukraine Perinatal Prevention of AIDS Initiative); Gary Murphy (Public Health England); Kholoud Porter, Anne Johnson, Andrew Phillips, Abdel Babiker (UK Register of HIV Seroconverters), Deenan Pillay (University College London, UK); Charles Morrison (Family Health International); Robert Salata (Case Western Reserve University); Roy Mugerwa (Makerere University, Uganda); Tsungai Chipato (University of Zimbabwe); Matt A. Price, Jill Gilmour, Anatoli Kamali (International AIDS Vaccine Initiative [IAVI] Early Infections Cohort); Etienne Karita (Projet San Francisco, Rwanda).

**EuroCoord Executive Board:** Fiona Burns, University College London, UK; Geneviève Chêne, University of Bordeaux, France; Dominique Costagliola (Scientific Coordinator), Institut National de la Santé et de la Recherche Médicale, France; Carlo Giaquinto, Fondazione PENTA, Italy; Jesper Grarup, Region Hovedstaden, Denmark; Ole Kirk, Region Hovedstaden, Denmark; Laurence Meyer, Institut National de la Santé et de la Recherche Médicale, France; Heather Bailey, University College London, UK; Alain Volny Anne, European AIDS Treatment Group, France; Alex Panteleev, St. Petersburg City AIDS Centre, Russian Federation; Andrew Phillips, University College London, UK, Kholoud Porter, University College London, UK; Claire Thorne, University College London, UK.

**EuroCoord Council of Partners:** Jean-Pierre Aboulker, Institut National de la Santé et de la Recherche Médicale, France; Jan Albert, Karolinska Institute, Sweden; Silvia Asandi, Romanian Angel Appeal Foundation, Romania; Geneviève Chêne, University of Bordeaux, France; Dominique Costagliola (chair), INSERM, France; Antonella d’Arminio Monforte, ICoNA Foundation, Italy; Stéphane De Wit, St. Pierre University Hospital, Belgium; Peter Reiss, Stichting HIV Monitoring, Netherlands; Julia Del Amo, Instituto de Salud Carlos III, Spain; José Gatell, Fundació Privada Clínic per a la Recerca Bíomèdica, Spain; Carlo Giaquinto, Fondazione PENTA, Italy; Osamah Hamouda, Robert Koch Institut, Germany; Igor Karpov, University of Minsk, Belarus; Bruno Ledergerber, University of Zurich, Switzerland; Jens Lundgren, Region Hovedstaden, Denmark; Ruslan Malyuta, Perinatal Prevention of AIDS Initiative, Ukraine; Claus Møller, Cadpeople A/S, Denmark; Kholoud Porter, University College London, United Kingdom; Maria Prins, Academic Medical Centre, Netherlands; Aza Rakhmanova, St. Petersburg City AIDS Centre, Russian Federation; Jürgen Rockstroh, University of Bonn, Germany; Magda Rosinska, National Institute of Public Health, National Institute of Hygiene, Poland; Manjinder Sandhu, Genome Research Limited; Claire Thorne, University College London, UK; Giota Touloumi, National and Kapodistrian University of Athens, Greece; Alain Volny Anne, European AIDS Treatment Group, France.

**EuroCoord External Advisory Board:** David Cooper, University of New South Wales, Australia; Nikos Dedes, Positive Voice, Greece; Kevin Fenton, Public Health England, USA; David Pizzuti, Gilead Sciences, USA; Marco Vitoria, World Health Organisation, Switzerland.

**EuroCoord Secretariat:** Silvia Faggion, Fondazione PENTA, Italy; Lorraine Fradette, University College London, UK; Richard Frost, University College London, UK; Andrea Cartier, University College London, UK; Dorthe Raben, Region Hovedstaden, Denmark; Christine Schwimmer, University of Bordeaux, France; Martin Scott, UCL European Research and Innovation Office, UK.

Ethics approval has been granted by the following committees:

Austrian HIV Cohort Study: Ethik-Kommission der Medizinischen Universität Wien, Medizinische Universität Graz – Ethikkommission, Ethikkommission der Medizinischen Universität Innsbruck, Ethikkommission des Landes Oberösterreich, Ethikkommission für das Bundesland Salzburg; PHAEDRA cohort: St Vincent's Hospital, Human Research Ethics Committee; Southern Alberta Clinic Cohort: Conjoint Health Research Ethics Board of the Faculties of Medicine, Nursing and Kinesiology, University of Calgary; Aquitaine Cohort: Commission Nationale de l'Informatique et des Libertés; French PRIMO Cohort: Comite Consultatif de Protection des Personnes dans la Recherché Biomedicale; German HIV-1 Seroconverter Study: Charité, University Medicine Berlin; AMACS: Bioethics & Deontology Committee of Athens University Medical School and the National Organization of Medicines; Greek Haemophilia Cohort: Bioethics & Deontology Committee of Athens University Medical School and the National Organization of Medicines; ICoNA cohort: San Paolo Hospital Ethic Committee; Amsterdam Cohort Studies in Homosexual Men: Academic Medical Centre, University of Amsterdam; Oslo and Oslo University Hospital Cohorts: Regional komite for medisinsk forskningsetikk - Øst- Norge (REK 1); CoRIS-scv: Comité Ético de Investigación Clínica de La Rioja; Madrid Cohort: Ethics Committee of Universidad Miguel Hernandez de Elche; Swiss HIV Cohort Study: Kantonale Ethikkommission, spezialisierte Unterkommission Innere Medizin, Ethikkommission beider Basel, Kantonale Ethikkommission Bern, Comité départemental d'éthique de médecine et médecine communautaire, Commission d'éthique de la recherche clinique, Université de Lausanne, Comitato etico cantonale, Ethikkommission des Kantons St.Gallen; UK Register of HIV Seroconverters: South Birmigham REC; Early Infection Cohorts: Kenya Medical Research Institute, Kenyatta National Hospital.
